# Of the Decussation of the Optic Nerves in Quadrupeds

**Published:** 1784

**Authors:** 


*Of the decuſſation of the optic nerves in quadrupeds.*

PROFESSOR Soemmering, of Caſſel, has lately had an opportunity of ſeeing the decuſſation of the optic nerves in ſome ſpecies of quadrupeds. As no anatomiſt, as far as I know, has ever obſerved it in animals of this kind, I have no doubt but a ſhort account of this diſcovery will prove agreeable to you; particularly as I have had an opportunity of examining all the anatomical preparations, on which the fact is eſtabliſhed.

The firſt animal in which the profeſſor obſerved this remarkable phenomenon, was a ſquirrel, which happened to have a cataract in its left eye. On opening the cranium with all poſſible care, he found the nerve of the affected eye more oval, flatter, in every reſpect ſmaller, and of a more cineritious colour, than the nerve of the healthy one; and this evident difference in the colour, ſhape, &c. of the affected nerve, might be clearly traced acroſs the union of the optic nerves, to the right ſide, while the ſound nerve might be ſeen croſſing this union, and afterwards appearing of its natural ſize and colour on the left ſide of the brain.

He ſoon after had occaſion to obſerve the ſame appearance in a horſe, whoſe right eye was ſmall and collapſed, while the left was in a healthy ſtate. In this animal the nerve of the affected eye was found to be ſhorter, ſmaller, of a cineritious colour, and nearly of a cartilaginous hardneſs, adhering firmly to its dura mater coat. All theſe appearances (the laſt one of courſe excepted) might be traced acroſs the union of the optic nerves from the right ſide to the left, in the ſame manner as had before been obſerved in the ſquirrel.

The profeſſor has ſeen nearly the ſame appearance in the brain of another horſe, but with this addition, that the diſeaſed nerve, which croſſed the other, was diſtinguiſhed by a cineritious, and, in ſome degree, pellucid ſtreak, which made a very ſtriking contraſt with the milk-white ſubſtance of the other ſound nerve, which which appeared at the place where they crofsed.

But ftill more interefting is the obfervation he made in a monftrous pig with two heads, each of which had a diftinct brain. In one of thefe heads there was no appearance of an eye or of a nerve belonging to it, on the interior or left fide, but the right eye was perfect; and the nerve of this eye, which was in a found ftate, evidently croffed over to the left fide, where it continued to retain its natural appearance.

From thefe obfervations, however, though extremely interefting, we cannot infer, that a fimilar organization takes place in the human body; and I am aware, that the illuftrious Morgagni has been unable to difcover any thing like it in the brain of blind men, and alfo that he has fought for it in vain in dogs. Petit affures us he has met with it in birds; in the *Cyclopterus Lumpus* it has been feen by the celebrated Camper; in the *Raja* and *Squalus acanthus*, by Profeffor Soemmering; and in fifhes by every anatomift. My ingenious friend fufpects, that all the nerves crofs one another in the more interior parts of the brain or medulla fpinalis; a conjecture which deferves to be the object of future experiments.

